# Effect of the NU-AGE Diet on Cognitive Functioning in Older Adults: A Randomized Controlled Trial

**DOI:** 10.3389/fphys.2018.00349

**Published:** 2018-04-04

**Authors:** Anna Marseglia, Weili Xu, Laura Fratiglioni, Cristina Fabbri, Agnes A. M. Berendsen, Agata Bialecka-Debek, Amy Jennings, Rachel Gillings, Nathalie Meunier, Elodie Caumon, Susan Fairweather-Tait, Barbara Pietruszka, Lisette C. P. G. M. De Groot, Aurelia Santoro, Claudio Franceschi

**Affiliations:** ^1^Department of Neurobiology, Care Sciences and Society, Aging Research Center, Karolinska Institutet and Stockholm University, Stockholm, Sweden; ^2^Department of Epidemiology and Biostatistics, School of Public Health, Tianjin Medical University, Tianjin, China; ^3^Stockholm Gerontology Research Center, Stockholm, Sweden; ^4^Department of Experimental, Diagnostic and Specialty Medicine, University of Bologna, Bologna, Italy; ^5^Division of Human Nutrition, Wageningen University & Research, Wageningen, Netherlands; ^6^Department of Human Nutrition, Warsaw University of Life Sciences - SGGW, Warsaw, Poland; ^7^Norwich Medical School, University of East Anglia, Norwich, United Kingdom; ^8^Centre Hospitalier Universitaire de Clermont-Ferrand, Clermont-Ferrand, France; ^9^C.I.G. Interdepartmental Centre “L. Galvani,” University of Bologna, Bologna, Italy; ^10^Institute of Neurological Sciences (IRCCS), Bologna, Italy

**Keywords:** randomized controlled trial, dietary intervention, cognitive decline, multicenter, neuroprotective, episodic memory, healthy diet

## Abstract

**Background:** Findings from animal and epidemiological research support the potential neuroprotective benefits from healthy diets. However, to establish diet-neuroprotective causal relations, evidence from dietary intervention studies is needed. NU-AGE is the first multicenter intervention assessing whether a diet targeting health in aging can counteract the age-related physiological changes in different organs, including the brain. In this study, we specifically investigated the effects of NU-AGE's dietary intervention on age-related cognitive decline.

**Materials and Methods:** NU-AGE randomized trial (NCT01754012, clinicaltrials.gov) included 1279 relatively healthy older-adults, aged 65–79 years, from five European centers. Participants were randomly allocated into two groups: “control” (*n* = 638), following a habitual diet; and, “intervention” (*n* = 641), given individually tailored dietary advice (NU-AGE diet). Adherence to the NU-AGE diet was measured over follow-up, and categorized into tertiles (low, moderate, high). Cognitive function was ascertained at baseline and at 1-year follow-up with the Consortium to Establish a Registry for Alzheimer's Disease (CERAD)-Neuropsychological Battery and five additional domain-specific single cognitive tests. The raw scores from the CERAD subtests [excluding the Mini-Mental State Examination (MMSE)] and the single tests were standardized into *Z*-scores. Global cognition (measured with MMSE and CERAD-total score), and five cognitive domains (perceptual speed, executive function, episodic memory, verbal abilities, and constructional praxis) were created. Cognitive changes as a function of the intervention were analyzed with multivariable mixed-effects models.

**Results:** After the 1-year follow-up, 571 (89.1%) controls and 573 (89.8%) from the intervention group participated in the post-intervention assessment. Both control and intervention groups showed improvements in global cognition and in all cognitive domains after 1 year, but differences in cognitive changes between the two groups were not statistically significant. However, participants with higher adherence to the NU-AGE diet showed statistically significant improvements in global cognition [β 0.20 (95%CI 0.004, 0.39), *p*-value = 0.046] and episodic memory [β 0.15 (95%CI 0.02, 0.28), *p*-value = 0.025] after 1 year, compared to those adults with lower adherence.

**Discussion:** High adherence to the culturally adapted, individually tailored, NU-AGE diet could slow down age-related cognitive decline, helping to prevent cognitive impairment and dementia.

## Introduction

Dementia has become a worldwide public health priority. About 47 million people are living today with dementia around the world and this number is expected to increase three-fold, up to 132 million cases by 2050 (Prince et al., 2015). Currently, treatment to cure dementia, or its preclinical stages, is not available and preventative strategies are still under debate. Lifestyle modification appears to be a promising alternative strategy to slow the progression of cognitive decline and thus, to prevent dementia (Winblad et al., 2016).

Emerging evidence from observational studies suggests that healthy dietary habits could have neuroprotective benefits. A slower rate of cognitive decline and reduced risk of cognitive impairment and dementia have been associated with a higher consumption of single nutrients (e.g., vitamins B–E, tocopherol, polyunsaturated omega-3 fatty acids, polyphenols; Mangialasche et al., 2010, 2013; Gillette-Guyonnet et al., 2013; Hooshmand et al., 2014) or of single foods (e.g., olive oil, vegetables, fruits, nuts, red wine, and fish; Devore et al., 2012; Caracciolo et al., 2014). However, people eat combinations of foods, which may have additive, synergistic, or antagonistic effects on health (Jacobs et al., 2009). Therefore, using a whole-diet approach, which emphasizes the overall dietary intake, is more important when seeking for preventive strategies targeting multifactorial disorders, such as dementia.

In the last half century, the Mediterranean diet, traditionally widespread across the Mediterranean Sea countries (e.g., Italy, Greece, Egypt, Libya), has been extensively investigated in relation to several health outcomes, making it a potential model of a healthy and sustainable diet (Dernini and Berry, 2015; Boccardi et al., 2018). The Mediterranean diet is characterized by a high intake of plant-based foods (i.e., vegetables, fruits, whole grain, cereals, legumes, nuts, and seeds); olive oil as the main source of fat; low-to-moderate consumption of dairy products, fish, and poultry; and low intake of red meat and red wine during meals (Boccardi et al., 2018). Greater adherence to the Mediterranean diet has been related to decreased risks of several age-related health conditions, such as metabolic, cardiovascular, and cerebrovascular disorders, cancer (Ostan et al., 2015), and mortality (Yannakoulia et al., 2015), but also cognitive decline (Trichopoulou et al., 2015), cognitive impairment (Psaltopoulou et al., 2013), and dementia (Scarmeas et al., 2006; Singh et al., 2014). Similar results were also observed in the few population-based studies which have used culturally adapted Mediterranean-like dietary patterns such as the MIND diet (Morris et al., 2015), the Prudent diet (Shakersain et al., 2016), and the Baltic Sea Diet (Kanerva et al., 2014).

Despite the encouraging findings from these observational studies, evidence from dietary intervention randomized controlled trials (RCTs) is lacking in healthy older adults, which is needed in order to establish diet-neuroprotective causal relationships. NU-AGE is the first multicenter whole-diet intervention assessing whether a diet targeting national nutritional recommendations for older adults can counteract inflammaging and age-related physiological changes in different organs, including the brain. Specifically, in the current study, we investigated the effects of NU-AGE's dietary intervention on cognitive functioning in relatively healthy older adults from five different European countries.

## Materials and methods

### Study design and participants

NU-AGE (http://www.nu-age.eu/) is a 1-year, multicenter, randomized, single-blind, controlled trial (registered with clinicaltrials.gov, NCT01754012) with two parallel groups (i.e., dietary intervention and control) carried out during April 2012–January 2015 in five European centers in France, Italy, the Netherlands, Poland, and the United Kingdom (UK) (Berendsen et al., 2014). The recruitment of participants has been described in detail previously (Berendsen et al., 2014; Santoro et al., 2014). Briefly, 2,668 volunteers from the community aged 65–79 years, free of major overt chronic diseases for at least 2 years (e.g., cancer, severe organ disease), living independently, and free of dementia, were recruited to participate in the baseline assessment. At enrollment, exclusion criteria included severe heart diseases, type 1 and insulin-treated type 2 diabetes, chronic use of corticosteroids, recent use of antibiotics, change in habitual medication use, frailty (Fried et al., 2001), malnutrition [body mass index (BMI) <18.5 kg/m^2^ or 10% weight loss within 6 months], or food allergy/intolerance requiring special diets. Of the 2,665 participants, 1512 were screened for inclusion and 1,296 were eligible to participate in the NU-AGE trial. In this study, 1,279 participants [intention-to-treat (ITT) population] who completed the baseline cognitive assessment were randomly assigned (1:1) to the control (*n* = 641) or intervention (*n* = 638) groups, after stratification by sex, age (65–72 or >72–79 years), frailty status (pre-frail or non-frail), and BMI (<25 or ≥25 kg/m^2^). Randomization was done by computer-generated allocation. Participants were informed about their study group after randomization.

Written informed consent was collected from all participants prior to their inclusion in the study, in accordance with the Declaration of Helsinki. NU-AGE was approved by the ethics committee of the coordinator center—the Independent Ethics Committee of the S. Orsola-Malpighi Hospital Bologna (Italy)—and by the local/national ethics committees of all the other four recruiting centers—the South-East 6 Person Protection Committee (France), the Wageningen University Medical Ethics Committee (Netherlands), the National Research Ethics Committee–East of England (UK), and the Bioethics Committee of the Polish National Food and Nutrition Institute (Poland).

### Procedures

Before recruitment started, dieticians, research nutritionists, and research assistants from the five recruiting centers participated in structured in-depth training for recruitment, data collection (e.g., how to administer questionnaires; assess nutritional, physical, and cognitive assessment; collect specimen samples), and dietary intervention. At baseline and 1-year of follow-up, trained personnel (i.e., research assistants, dieticians, or research nutritionist), who were blinded to the study group, collected information on demographics (i.e., age, sex, education), medical conditions, and medication use at each research center through structured interviews and questionnaires. Among several parameters collected (Santoro et al., 2014), weight, height, and blood pressure were measured by a trained research assistant. Blood, urine, and fecal samples were collected, handled, and stored from all participants. Education was categorized into elementary, secondary (including lower and upper secondary), and college/university based on years of full-time education. Weight and height were measured while participants wore light clothes and no shoes. Country of enrollment was categorized into “France,” “Holland,” “Italy,” “Poland,” and “UK.” BMI was calculated as weight in kilograms divided by squared height in meters (kg/m^2^) and classified as underweight (<20), normal weight (20–25), overweight (25–30), and obese (≥30). Frailty was based on the five criteria proposed by Fried and colleagues (Fried et al., 2001), including weight loss, weakness (i.e., poor handgrip strength), self-reported exhaustion, slowness (i.e., slow gait speed), and low physical activity. Weight loss was defined as self-reported unintentional loss (i.e., not due to diet or physical exercise) of ≥4.5 kg in the last 12 months. Handgrip strength was measured three times in the dominant hand using the Scandidact Smedley's Hand Dynamometer® (Odder, Denmark). Weakness was defined as the average handgrip strength equal or below the sex- and BMI-specific cutoffs provided by Fried and colleagues (Fried et al., 2001). Two questions from the Center for Epidemiologic Studies Depression (CES-D) scale were administered as measures of exhaustion: “I felt that everything I did was an effort” and “I could not get going” (Gray et al., 2013; Sousa-Santos et al., 2018). Self-reported exhaustion was present if at least one condition was present for ≥3 days in the past week. Gait speed was measured by asking participants to walk at their usual speed over 4.6 m (Sousa-Santos et al., 2018). Slow gait was defined as walking equal to or above the sex- and height-specific validated cutoffs (Fried et al., 2001). Physical activity was determined based on weekly rates of energy expenditure (kcal/week), derived from the modified Minnesota Leisure Time Activity Questionnaire, in which participants were asked about frequency and duration of time spent in 18 activities over the past 2 weeks (Taylor et al., 1978; Siscovick et al., 1997). Low physical activity was defined as <383 kcal for men or <270 kcal for women, according to sex-specific cutoffs (Fried et al., 2001). Pre-frailty was defined as the presence of 1 or 2 of the above criteria. Medical conditions were self-reported, but verified, when possible, by the recruiting staff by asking the participants to bring their medical documents and current drug boxes to the visit. In some cases, the staff was authorized by the participants to contact their general practitioner for further health information. Medical conditions included sensory disorders (e.g., impaired vision, hearing, olfactory, or taste), hypertension, hypercholesterolemia, cardiovascular and cerebrovascular diseases, neurological and mental health issues, thyroid dysfunction, osteoporosis, and arthritis.

### Dietary intervention and controls

Details on the dietary intervention, control group, and dietary intake assessments were reported previously (see Berendsen et al., 2014). In brief, all participants (randomized into control and intervention groups) completed a seven-day food record for seven consecutive days over 2 weeks after the baseline assessment, and had an interview with a trained dietician/research nutritionist to review the records. The consumed foods recorded in the food record were coded according to standardized coding procedures (Berendsen et al., 2014). The control group, following a habitual diet, received only a leaflet with national dietary guidelines. The intervention group followed the NU-AGE diet, which consisted of individually tailored Mediterranean-like diet advice targeting dietary recommendations for older adults from the five included countries (see Berendsen et al., 2014 for more information on the NU-AGE nutritional reccomandations). Participants in the diet group also received individual education by means of monthly telephone contacts and face-to-face meetings with a dietician/nutritionist. In particular, at months 4 and 8 the dietician/nutritionist used the Motivational Interviewing technique during nutritional counseling to help supporting participants to meet the NU-AGE Food Based Dietary Guidelines (FBDG). Additionally, to increase adherence, participants in the intervention group received free food, meeting the NU-AGE guidelines [e.g., whole grain pasta, margarine rich in PUFA and MUFA, low fat, low-salt cheese, extra virgin olive oil and frozen vegetable soup (Italy only)]. A NU-AGE index was constructed to measure the participant's level of adherence to the to the NU-AGE's FBDG, both at baseline and post-intervention (details on the adherence indices are reported in Berendsen et al., under review).

### Assessment of cognitive functioning

A comprehensive cognitive test battery was administered by trained personnel (who were blinded to the participant's assigned group), in a fixed order, at baseline and 1-year of follow-up. The battery included the Consortium to Establish a Registry for Alzheimer's Disease neuropsychological battery [CERAD-NB, which included the Mini Mental State Examination (MMSE)] (Morris et al., 1989), Babcock Story Recall Test (BSRT) (Babcock and Levy, 1940), Pattern Comparisons (Salthouse and Babcock, 1991), Digit Cancellation (Zazzo, 1974), and Trail Making Test (TMT) (Lezak et al., 2012). These tests covered the major cognitive functions and were sensitive enough to detect small-to-moderate differences in cognition in normal aging (Lezak et al., 2012).

The Mini-Mental State Examination (MMSE) is a 30-point screening tool, commonly used in clinical and research settings to assess global cognitive function (Folstein et al., 1975; Lezak et al., 2012). Due to its global nature and being a brief cognitive screening tool, the MMSE was used on its own in the current study (Chandler et al., 2005). Therefore, the CERAD-NB consisted of seven subtests. The *Category fluency* task required participants to name as many animals as possible and is scored as the number of correct unique words generated in 60 s. In the *15-items Boston Naming Test* (15-BNT), participants were instructed to name 15 objects, ranging in frequency of occurrence (low, medium, or high), presented as line drawings in a flipbook. The number of correctly named objects was recorded. The *Word List Memory* (WLM) test consisted of three tasks: immediate and delay recall, and recognition. Participants were shown ten consecutive and unrelated words over three trials and instructed to read each word aloud and then to remember as many words as possible (*immediate* recall). The total score was the sum of items correctly recalled over the three trials. After approximately 5 min, participants were asked to recall the 10 words, recording the maximum number of correct responses (*delay* recall). Next, participants were asked to recognize the 10 words from a list of targets mixed with unrelated distractors (*recognition*). The recognition score was calculated as the number of correct hits minus the number of false alarms (Laukka et al., 2013). The *Constructional Praxis test* instructed participants to draw four geometrical figures increasing in their complexity; the score was the sum of the correct responses on the four figures (range 0–11).

In the BSRT (Babcock and Levy, 1940) participants were read a short story consisting of an immediate and a delayed (after 20 min) recall of a 21-unit paragraph presented in an organized context. The Pattern comparisons test (Salthouse and Babcock, 1991) was scored as the number of correct classifications—“same” or “different”—of pairs of line-segment patterns. For the Digit Cancellation test (Zazzo, 1974), participants were instructed to cross every “4” encountered among random digits; the score was the number of correct 4s crossed in 30 s. TMT (Lezak et al., 2012) is a paper-and-pencil test consisting of two similar parts, A and B. The TMT-A requires the sequential connecting of 25 circled numbers, while in the TMT-B the participants were instructed to sequentially alternate numbers and letters (e.g., 1, A, 2, B). For each part, the scores (time of performance in seconds) were multiplied by −1, therefore higher scores always indicated better performance. A TMT B/A ratio score was generated as a measure of executive function independent of the motor speed component (Lezak et al., 2012).

The MMSE and a CERAD total score (CTS), which was calculated by summing the CERAD-NB subtest scores, were used as measures of global cognition (Chandler et al., 2005). Further, at each time point, raw scores from the cognitive tests were standardized into Z-scores, using baseline means and standard deviations (SDs). Five cognitive domains were created using individual test Z-scores or by averaging the Z-scores of multiple tests. The division was made a priori, according to the standard neuropsychological practice and cognitive theory (Lezak et al., 2012). Final cognitive domains included: (1) Perceptual speed [Pattern comparisons, Digit cancellation]; (2) Executive functions [Category fluency, TMT B/A ratio]; (3) Episodic memory, resulting from the sum of “immediate” [WLM and BSRT immediate tasks] and “delay” [WLM and BSRT delayed tasks] recall; (4) Verbal abilities (15-BNT); and (5) Constructional praxis (CERAD copy task). An overview of the cognitive assessment included in NU-AGE is provided in Table 1.

**Table 1:** Overview of the cognitive domains and related tasks in the NU-AGE study.

**Cognitive domains**	**Cognitive tasks**
Global cognition	Mini-Mental State Examination
	CERAD total score^a^
Perceptual speed^b^	Pattern comparisons
	Digit cancellation
Executive function^b^	Category fluency
	Trial making test B/A ratio
Episodic memory^b^	World List Memory—immediate and delay recall
	Babcock Story Recall Test—immediate and delay recall
Verbal abilities^b^	15-items Boston Naming Test
Constructional praxis^b^	Constructional praxis test

a *CERAD total score was calculated by summing the z-scores from the following CERAD subtests: Category fluency; 15-items Boston Naming Test; World List Memory—immediate and delay recall; World List Memory—recognition task; and, Constructional praxis test*.

b *Composite cognitive domains were created by averaging the z-scores of the cognitive tasks reported in the second column. Constructional praxis was based on the individual praxis test z-score*.

### Statistical analysis

Baseline characteristics between groups were analyzed using chi-square tests for proportions, and *t*-tests, or one-way ANOVAs for continuous variables, followed by pairwise comparisons with Bonferroni correction. Linear mixed-effects models, with maximum likelihood estimation and identity covariance matrices, were used to assess mean global and domain-specific cognitive changes between groups 1-year after randomization. Each cognitive measure was used as a separate outcome. The fixed effects included group (control vs. intervention), linear time (baseline vs. 1-year follow-up), and their interaction (group × time). The random effects included random intercept and slope for time, allowing for intra-individual differences in cognitive performance at baseline and over time. Mixed-effects models were also employed to examine the association between adherence to NU-AGE diet and cognitive changes within the intervention group. The NU-AGE index was divided into tertiles (low, moderate, high), with the lowest tertile (lowest adherence) being used as the reference category. Baseline age, sex, education, enrollment country, interviewer, baseline adherence in tertiles, BMI, and medical conditions were considered as potential confounders. The primary analyses were based on the ITT population.

In supplementary analyses, we conducted modified intention-to-treat analysis (mITT), which included participants with complete cognitive data at baseline and the follow-up examination. Country-related differences in cognitive changes over 1-year of follow-up were investigated in stratified analyses by enrollment country. We further performed stratified analyses by baseline (1) cognitive functioning (cognitive impairment [MMSE <27] vs. cognitively intact [MMSE ≥27]); (2) frailty status; and 3) educational attainment [low (primary plus lower secondary) vs. high (upper secondary plus college)].

Two-tailed *p* values ≤ 0.05 were deemed statistically significant in all analyses. Stata SE, version 14.0 (StataCorp LP., College Station, Texas, USA) was used in all data analyses.

## Results

### Characteristics of study participants

At baseline, the average age of the 1,279 participants was 70.9 ± 3.4 years, and 720 (56.3%) were female. Each center recruited a similar proportion of participants [France, *n* = 210 (16.4%); the Netherlands, *n* = 252 (19.7%); Italy, *n* = 298 (23.3%); Poland, *n* = 252 (19.7%); and UK, *n* = 267 (20.9%)]. Baseline demographic, health-related, and cognitive characteristics of study participants were overall balanced (Table 2, Supplementary Table 1). However, participants in the intervention group were more likely to be slightly younger, pre-frail, and to have a higher proportion of mental health conditions. After 1 year, 1,144 (89.4%) participated at the follow-up assessment, of whom 571 (89.1%) were controls and 573 (89.8%) were from the intervention group. Both control and intervention groups tended to have improvements in global cognition and in all cognitive domains, but differences were not statistically significant between groups (Supplementary Table 1).

**Table 2:** Baseline demographics and health characteristics of NU-AGE study participants (*n* = 1279).

**Characteristics**	**No. participants with available information**	**Control**	**Intervention**	***p*-value^a^**
		***n* = 641**	***n* = 638**	
Age, years	1279	71.1 ± 0.2	70.7 ± 0.2	0.052
Female sex	1279	353 (55.1)	367 (57.5)	0.376
Education	1242			
Elementary		25 (4.0)	16 (2.6)	
Secondary		303 (49.0)	301 (48.3)	0.309
College/University		291 (47.0)	306 (49.1)	
Country	1279			
France		105 (16.4)	105 (16.5)	
Holland		129 (20.1)	123 (19.3)	
Italy		152 (23.7)	146 (22.9)	0.981
Poland		124 (19.3)	128 (20.1)	
UK		131 (20.4)	136 (21.3)	
Baseline adherence^b^	1232	51.5 ± 10.1	51.5 ± 9.56	0.956
MMSE	1243	28.3 ± 1.59	28.2 ± 1.68	0.188
Frailty status	1142			
Non-frail		466 (81.8)	434 (75.9)	0.015
Pre-frail		104 (18.3)	138 (24.1)	
HbA1c, %	1227	5.8 ± 0.02	5.8 ± 0.02	0.835
Body-mass index, kg/m^2^	1277	26.7 ± 0.1	26.8 ± 0.2	0.613
Underweight [<20]		13 (2.0)	9 (1.4)	
Normal [20-25]		201 (31.5)	225 (35.3)	
Overweight [25-30]		304 (47.6)	280 (43.9)	0.376
Obese [≥30]		121 (18.9)	124 (19.4)	
**MEDICAL CONDITIONS**				
Sensorial	1244	357 (57.6)	343 (55.0)	0.353
Hypertension	1245	249 (40.2)	265 (42.4)	0.422
Hypercholesterolemia	1241	206 (33.4)	205 (32.9)	0.841
Cardiovascular diseases	1245	180 (29.0)	190 (30.4)	0.598
Stroke	1245	12 (1.9)	13 (2.1)	0.856
Diabetes	1245	33 (5.3)	30 (4.8)	0.674
Neurological	1244	14 (2.3)	19 (3.0)	0.388
Mental health	1241	26 (4.2)	44 (7.0)	0.030
Hyperthyroid	1244	4 (0.7)	9 (1.4)	0.169
Hypothyroid	1244	58 (9.4)	63 (10.1)	0.673
Osteoporosis	1239	68 (11.0)	79 (12.7)	0.372
Arthritis	1238	193 (31.4)	202 (32.4)	0.723

*Data are presented as proportion [n (%)] or mean ± standard deviations*.

*HbA1c, glycated hemoglobin; MMSE, Mini-Mental State Examination*.

a *Two–sample t–test (p-value < 0.05)*.

b *Baseline adherence measures the participant's level of adherence to the NU-AGE's FBDG (details reported in Berendsen et al., under review)*.

During the 1-year intervention, 135 individuals withdrew from the study, because of the inability to follow the diet, difficulties to attend the 12-months visit, restrictiveness of dietary intervention, or issues of health or in the family. Compared to participants, dropouts had lower education, higher BMI, more sensorial impairment, cardiovascular diseases, osteoporosis, and lower cognitive function (mean MMSE score was 27.8 ± 2.0 for dropouts vs. 28.3 ± 1.6 for participants) at baseline. The highest proportion of dropouts were shown among participants in Italy (*n* = 56, 41.5%).

### Effects of NU-AGE intervention on cognition

**Figure 1:**
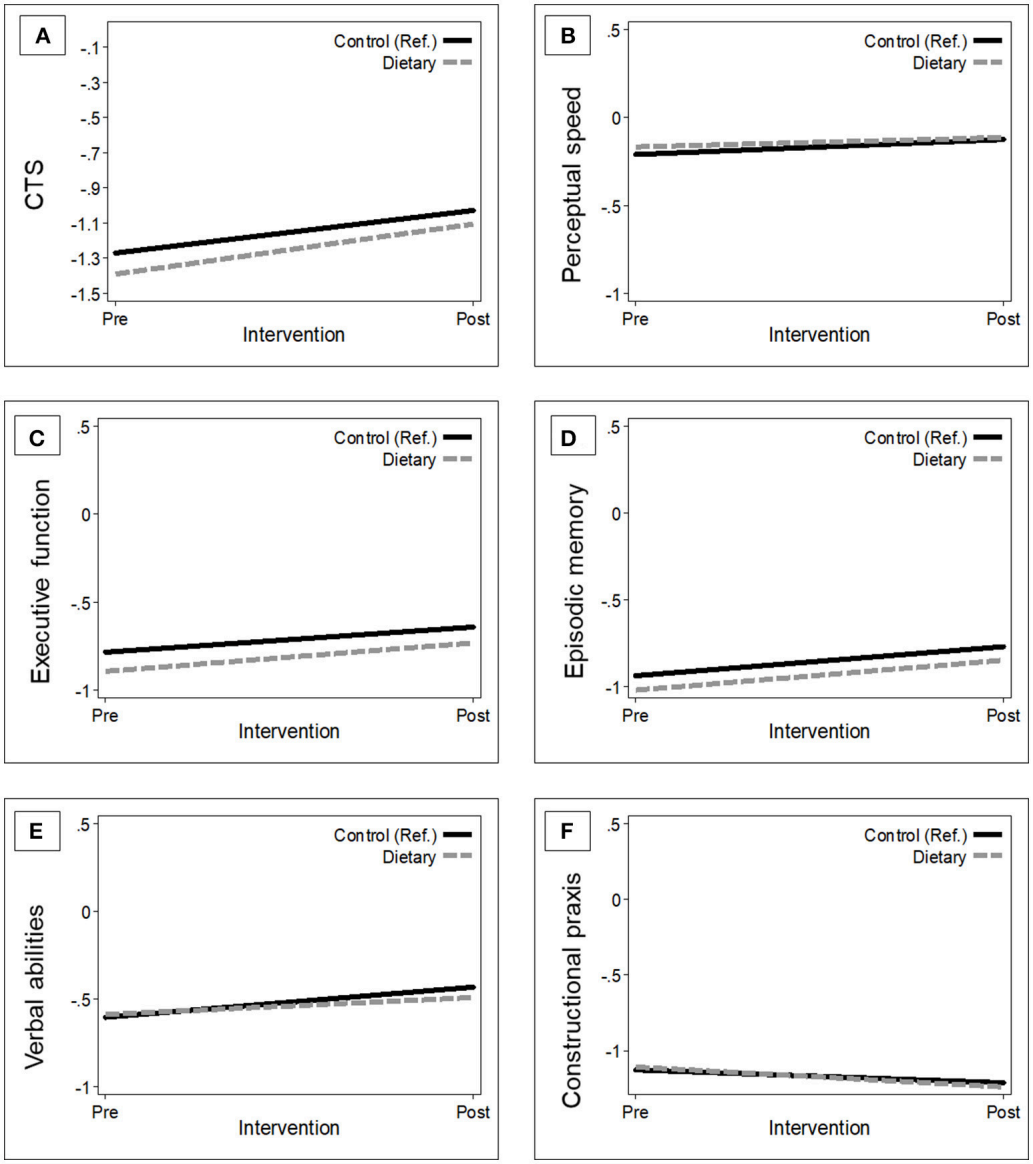
Estimated trajectories of change in global and domain-specific cognitive function 1-year after randomization by group. The figure shows the changes in global cognition **(A)**, perceptual speed **(B)**, executive functions **(C)**, episodic memory **(D)**, verbal abilities **(E)**, and constructional praxis **(F)** in the control group [*n* = 641; (reference); black solid line] vs. intervention group (*n* = 638; gray dash line). Mixed-effect models were adjusted for baseline age, sex, education, country, and interviewer. The trajectories were plotted using the mean values of the covariates. CTS, CERAD total score.

Overall, the MMSE score of participants in the intervention group improved by a mean of 0.15 points (95% CI −0.004, 0.31) in 1 year, whereas the mean MMSE change in controls was 0.11 points (95% CI −0.05, 0.27). However, this difference [0.04 (95% CI −0.17, 0.26)] was not statistically significant (*p* = 0.681). The basic-adjusted (by baseline age, sex, education, enrollment country, and interviewer) trajectories of global and domain-specific cognitive changes, 1 year after NU-AGE dietary intervention in the ITT population, are presented in Figure 1.

Participants in the intervention group showed a tendency toward improved performance on the CTS and in executive function compared to controls at 1-year follow-up. However, the 1-year changes in the mean differences for the CTS and the Z-scores of the cognitive domains were not statistically significant (Table 3). Results remained similar after fully-adjusting for baseline age, sex, education, enrollment country, interviewer, pre-frailty, BMI, and medical conditions (data not shown).

**Table 3:** Estimated change in global and domain-specific cognitive functioning 1-year after randomization in control (*n* = 641) and intervention (*n* = 638) groups.

**Global cognition**	β**-Coefficient (95% confidence interval)**^a^		***p*-value^b^**
	**Control**	**Intervention**	
**CTS**			
Baseline	1.98 (1.07; 2.90)	−0.12 (−0.22; −0.02)	0.020
Group × time	0.24 (0.18; 0.30)	0.04 (−0.04; 0.13)	0.321
**Cognitive domains**			
Perceptual speed			
Baseline	2.73 (2.02; 3.44)	0.04 (−0.3; 0.12)	0.263
Group × time	0.08 (0.04; 0.13)	−0.03 (−0.09; 0.03)	0.314
Executive functions			
Baseline	1.12 (0.43; 1.80)	−0.11 (−0.19; −0.03)	0.010
Group × time	0.14 (0.08; 0.21)	0.02 (−0.07; 0.11)	0.698
Episodic memory			
Baseline	1.13 (0.39; 1.86)	−0.08 (−0.16; −0.003)	0.042
Group × time	0.17 (0.12; 0.21)	0.004 (−0.06; 0.07)	0.890
Verbal abilities			
Baseline	2.27 (1.35; 3.20)	0.02 (−0.09; 0.12)	0.773
Group × time	0.17 (0.10; 0.24)	−0.07 (−0.17; 0.02)	0.109
Constructional praxis			
Baseline	0.10 (−0.74; 0.94)	0.02 (−0.08; 0.12)	0.688
Group × time	−0.08 (−0.17; −0.001)	−0.05 (−0.16; 0.06)	0.396

*CTS, CERAD total score; MMSE, Mini-Mental State Examination*.

a *Mixed-effect models were adjusted for baseline age, sex, education, center, and interviewer*.

b *p-value < 0.05*.

### NU-AGE diet adherence and cognitive improvements

**Figure 2:**
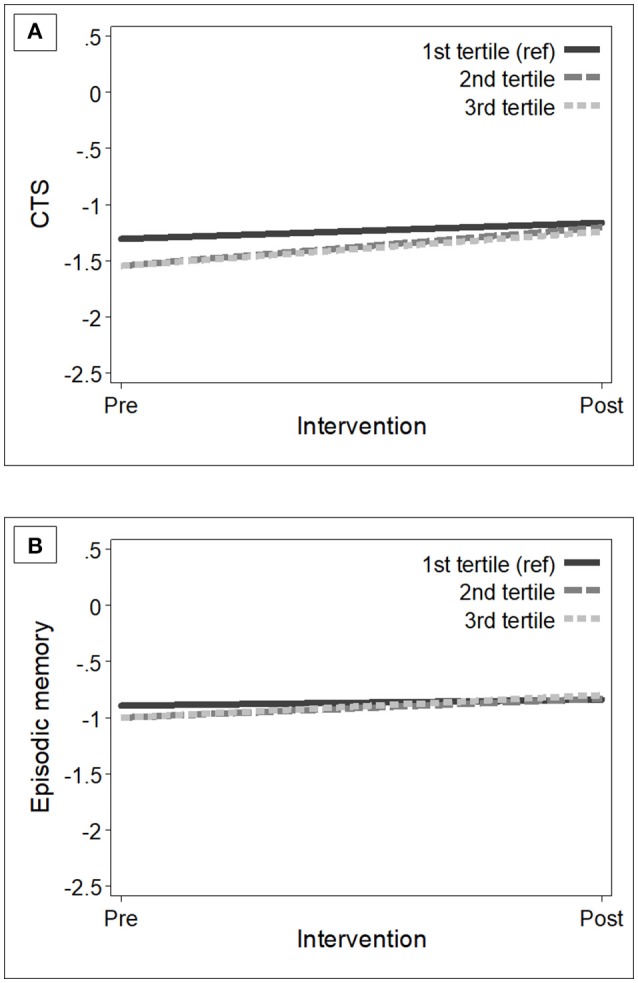
Estimated trajectories of change in global cognition **(A)** and episodic memory **(B)** among participants in the intervention group (*n* = 638) with low (first tertile, reference) to high (third tertile) adherence levels to the NU-AGE dietary intervention. Mixed-effect models were adjusted for baseline age, sex, education, country, and interviewer. The trajectories were plotted using the mean values of the covariates. CTS, CERAD total score.

At baseline, participants in the control and intervention groups showed similar dietary habits (measured with adherence to the NU-AGE's FBDG) (Table 2). As expected, after 1-year intervention, participants in the intervention group had increased levels of adherence (mean NU-AGE index = 65.9%, *SD* = 11.0) than those in the control group (mean NU-AGE index = 52.7%, SD = 10.0) [*p*-value < 0.001]. Within the intervention group, individuals with higher adherence to the NU-AGE diet [second tertile (ranged 53.3–64.3) and those in the third tertile (ranged 64.4–96.3)] showed statistically significant cognitive improvements in global cognition [β 0.20 (95% CI 0.004, 0.39), *p*-value = 0.046] and episodic memory [β 0.15 (95% CI 0.02, 0.28), *p*-value = 0.025] over the follow-up, compared to older adults with lower adherence [first tertile (ranged 25.4–53.3)] (Table 4). Fully adjusting for covariates did not alter the results. Figure 2 shows the estimated trajectories of cognitive change in the CTS and in episodic memory by adherence levels to the NU-AGE diet in the intervention group.

**Table 4:** Estimated mean change in global and domain-specific cognitive functioning (β-coefficients) and 95% confidence intervals by tertiles [1 (reference) to 3, corresponding to low to high adherence] to the NU-AGE diet over 1-year of follow-up, among the intervention group (*n* = 638).

**Cognitive functions**	**Adherence to the NU-AGE diet**		
	**1st tertile (low) β (95% CI)^a^**	**2nd tertile β (95% CI)^a^**	**3rd tertile (high) β (95% CI)^a^**
**CTS**			
Baseline	1.66(0.27, 3.05)	−0.24(−0.49, 0.01)	−0.24(−0.48, −0.003)^b^
Adherence × time	0.14(−0.02, 0.31)	0.20(0.004, 0.39)^b^	0.16(−0.02, 0.35)^c^
**Perceptual speed**			
Baseline	3.21(2.16, 4.28)	0.06(−0.12, 0.24)	0.07(−0.10, 0.24)
Adherence × time	0.09(−0.03, 0.20)	−0.04(−0.18, 0.09)	−0.04(−0.17, 0.08)
**Executive functions**			
Baseline	1.81(0.73, 2.91)	−0.06(−0.28, 0.15)	−0.14(−0.34, 0.07)
Adherence × time	0.19(−0.01, 0.38)	0.04(−0.18, 0.27)	−0.05(−0.26, 0.16)
**Episodic memory**			
Baseline	0.70(−0.45, 1.85)	−0.11(−0.31, 0.10)	−0.11(−0.30, 0.08)
Adherence × time	0.05(−0.07, 0.17)	0.12(−0.02, 0.26)	0.15(0.02, 0.28)^b^
**Verbal abilities**			
Baseline	2.56(1.17, 3.95)	−0.07(−0.33, 0.18)	−0.001(−0.24, 0.24)
Adherence × time	0.02(−0.15, 0.20)	0.11(−0.10, 0.31)	0.07(−0.13, 0.26)
**Constructional praxis**			
Baseline	0.17(−1.06, 1.40)	−0.06(−0.29, 0.18)	0.03(−0.20, 0.25)
Adherence × time	−0.22(−0.44, 0.01)	0.15(−0.12, 0.42)	0.07(−0.18, 0.32)

*CTS, CERAD total score*.

a *Mixed-effect models were adjusted for baseline age, sex, education, enrollment country, and interviewer*.

b *p-value < 0.05*.

c *p-value = 0.08*.

### Supplementary analyses

Basic- and fully-adjusted mixed-effects models were repeated in the mITT population, in supplementary analyses, which included participants with complete cognitive data at baseline and at follow-up (*n* = 1144). The effect magnitude of the NU-AGE dietary intervention on cognitive functioning was similar to the initial analyses. We also compared controls with low adherence to the FBDG (reference) vs. participants with moderate-to-high adherence to the NU-AGE diet. Compared to individuals in the control group with low adherence to the FBDG, participants with moderate-to-high adherence to the NU-AGE diet showed slight improvements in global cognition [β 0.06 (95% CI−0.04, 0.17)] and episodic memory [β 0.04 (95% CI −0.04, 0.11)] over the follow-up. However, these differences were not statistically significant.

Potential country-related differences in cognitive changes over the 1-year follow-up were assessed. We found significant differences in baseline demographic, health-related, and cognitive characteristics between the five enrollment countries (Supplementary Table 2). Therefore, a potential interaction was examined by incorporating a three-way product term [country × study groups (control and intervention) × time)] in the mixed-effects models, and by stratifying by enrollment country and study group. Results showed no significant interaction between enrollment country and test group. We investigated further potential interactions between test groups (control and intervention) and baseline factors such as cognitive impairment (cognitively healthy vs. cognitive impairment); pre-frail status (pre-frail vs. no frail); and educational attainment (low vs. high) in predicting cognitive changes over the 1-year follow-up in separate mixed-effect models. In stratified analyses, the associations between global/domain-specific cognitive changes and test group were not statistically different among participants with and without baseline cognitive impairment, pre-frailty, or low education. Therefore, educational attainment, enrollment country, baseline cognitive status, and pre-frail status did not alter the effects of the NU-AGE dietary intervention on cognitive function.

We repeated basic- and fully-adjusted mixed-effects models by excluding participants with neurological and mental health conditions at baseline (*n* = 103) and results were similar to the initial analyses (data not shown).

Finally, all analyses were repeated using a second CERAD total score, which was created by summing the seven CERAD-NB scores from category fluency, 15-BNT, WLM (immediate and delay recall, and recognition), and constructional praxis. Results were identical to the original analyses (using CTS; data not shown).

## Discussion

To the best of our knowledge, NU-AGE is the first multicenter RCT assessing the effect of a culturally adapted, individually tailored, Mediterranean-like whole-diet (dietary intervention vs. control groups) on global cognition and specific cognitive domains among relatively healthy older adults. In this study, we found that all participants showed improvements in their cognitive performance over 1-year follow-up, but no additional diet-related cognitive improvements were evident in the intervention arm. However, participants in the intervention group with the higher adherence to the NU-AGE diet had significantly improved global cognition, in particular episodic memory, after the intervention. Our findings suggest that a greater adherence to the individually tailored NU-AGE diet might slow cognitive decline, and thereby may help to prevent cognitive impairment and dementia.

To our knowledge, only three RCTs (PREDIMED, ENCORE, and MedLey) have previously assessed the effects of a Mediterranean dietary pattern on cognition in older adults in Mediterranean and non-Mediterranean countries (Smith et al., 2010; Valls-Pedret et al., 2015; Knight et al., 2016b). In the PREDIMED (Prevención con Dieta Mediterránea), 578 Spanish older adults, aged 55–80 years and at-risk of cardiovascular diseases (CVD), were randomly assigned to three arms (intervention groups: Mediterranean diet plus supplemented olive oil and Mediterranean diet plus supplemented nuts; control: standard low-fat diet) and were followed for 5 years. Participants allocated in the intervention groups showed cognitive improvements in response to the Mediterranean dietary pattern (Valls-Pedret et al., 2015). Furthermore, the 38 American older participants at-risk of CVD in the ENCORE (Exercise and Nutrition Interventions for Cardiovascular Health) trial showed slight improvements in psychomotor speed after 4-months intervention with the blood pressure-lowering Mediterranean-like DASH diet (emphasizing dairy consumption and restricted intake of sodium, commercial sweets, and saturated fat; Morris, 2016), compared with 43 controls who maintained their usual dietary habits (Smith et al., 2010). Conversely, no cognitive improvements were detected in 70 Australian healthy older volunteers, who followed a Mediterranean diet for 6 months (Knight et al., 2016b). In the NU-AGE trial, participants in the individually tailored dietary intervention arm showed a tendency toward global cognitive improvements after the 1-year intervention, but these changes were not significant when compared to the control group. Discrepancies in findings might be attributed to methodological differences related to the study sample (e.g., non-healthy vs. healthy older adults; small sample size), not high enough average adherence to diet in the whole intervention group, or, in particular, to the short duration of the dietary intervention. Indeed, the previous cohort studies have observed neurocognitive benefits over longer periods of time (up to 13 years; Féart et al., 2009; Morris et al., 2015; Trichopoulou et al., 2015).

Our findings are in line with previous community-based observational studies linking higher adherence to a Mediterranean diet or a similar dietary patterns with decreased cognitive decline (Solfrizzi et al., 2017). Greater adherence to a traditional Mediterranean diet was associated with lower cognitive decline over 5-to-8 years of follow-up in two prospective Mediterranean cohorts of older adults in France (Féart et al., 2009) and Greece (Trichopoulou et al., 2015). Similar results were also observed in non-Mediterranean countries. Tangney and colleagues (Tangney et al., 2011, 2014) reported slower rates of cognitive decline associated with a greater adherence to a Mediterranean diet in two cohorts of older individuals. A few studies in non-Mediterranean countries using culturally adapted healthy diets, based on the Mediterranean diet model, have reported similar relations. Recent findings from the MIND diet, which combined the Mediterranean and DASH diets with emphasis on green leafy vegetables and berries, showed reduced rates of cognitive decline in older individuals with increasing scores in the MIND diet (Morris et al., 2015). In Northern Europe, higher adherence to a Prudent diet (similar to the Mediterranean dietary pattern) was related to less cognitive decline over 6 years in a cohort of Swedish older adults (Shakersain et al., 2016). Interestingly, the authors observed that a more frequent intake of the Prudent diet, regardless of combining it with a less healthy Western diet (characterized by more red/processed meat, saturated/trans-fat, refined grains, sugar, beer, and spirit consumption), might still have neuroprotective benefits (Shakersain et al., 2016). Despite these positive findings, other observational studies did not detect significant associations between Mediterranean-like dietary patterns and cognitive health neither in Mediterranean countries (Psaltopoulou et al., 2008) nor in other countries (Cherbuin and Anstey, 2012; Samieri et al., 2013a,b). These inconsistent findings can be attributed to differences in the study population (e.g., inclusion of incident dementia or cognitive impairment), assessment of dietary intake (self-reported questionnaire, diaries, or more objective but less specific biomarkers) or dietary patterns (a posteriori vs. a priori), assessment of cognitive decline (screening tools such as MMSE rather than cognitive battery), or variation in baseline age of study populations and follow-up duration.

We also showed that a greater adherence to the NU-AGE diet was related, in particular, to improvements in episodic memory performance after intervention. Episodic memory impairment, a core feature of the typical Alzheimer's disease-related dementia (AD) phenotype, generally depends on large-scale brain networks of the medial temporal lobe, which includes the hippocampus (Dickerson and Eichenbaum, 2010; Dubois et al., 2010). In recent neuroimaging studies, higher adherence to Mediterranean-like diets was related to larger cerebral and hippocampal volumes over a period of up to 4-years (Gu et al., 2015; Jacka et al., 2015), supporting the potential neuroprotective benefits of healthy diets.

The mechanisms underlying the neuroprotective benefits linked to Mediterranean-like dietary patterns are largely unknown; however, some hypotheses have been put forward. At the systemic level, Mediterranean-like dietary patterns may improve cognition indirectly through cardiovascular improvements (Valls-Pedret et al., 2015). At the biochemical level, healthy dietary patterns are associated with decreased levels of oxidative stress, inflammatory biomarkers (Martucci et al., 2017), insulin resistance, and blood glucose (Knight et al., 2016a). Therefore, it has been proposed that synergistic interactions between inflammatory, oxidative stress-related and metabolic mechanisms may underlie the neuroprotective benefits of healthy dietary patterns (Vitale et al., 2013). Foods, such as fruits (e.g., berries, grapes, and nuts), vegetables (e.g., dark-leaf vegetables), whole grains, legumes/beans, fish (e.g., salmon, sardines, herring, tuna), and olive oil are rich in several potent dietary nutrients with antioxidant properties (vitamins A–E, omega-3 fatty acids, MUFAs, and flavonoids; Knight et al., 2016a). Higher intake of antioxidant nutrients is associated with reduced oxidative stress and neuro-inflammation, which are two key biological mechanisms of cognitive and brain aging (Fusco et al., 2007; Liu et al., 2017). Deficiency in antioxidants, overproduction of free radicals (e.g., reactive oxygen species, ROS), and/or ROS accumulation in the cells, might induce cellular oxidative damage, which over time leads to apoptotic neuronal death and, thus, to accelerated cognitive decline and dementia (Fusco et al., 2007; Calabrese et al., 2017; Liu et al., 2017). Furthermore, ROS accumulation can activate, directly or indirectly, specific proteins that trigger neuro-inflammation (Vitale et al., 2013). Chronic neuro-inflammation is associated with impaired microglia phagocytic capacity and astrocyte dysfunction—two major sources of increased concentration of inflammatory cytokines (e.g., TNFα, interlukine 1)—favoring amyloid-beta (Aβ) deposits, decreasing cerebral blood flow, and consequently, impairing adult neurogenesis (Heneka et al., 2015; Knight et al., 2016a). Animal studies can provide further understanding of the biochemical or structural brain effects related to specific types of diet, such as Mediterranean-like diets, thus partially explaining the observed cognitive improvements. In a study investigating the lipid profile and neuropeptidase activities in the cortex of rats fed with foods supplemented in fatty acids, particularly fish oil (rich in PUFA) or olive oil (rich in MUFA), results showed that the distribution of fatty acids in the cortex reflected the diet composition, which in turn influenced brain functioning (Segarra et al., 2011). The authors suggested that the lipids in the diet (especially higher concentration of omega-3 PUFAs) may change the membrane fluidity, which in turn will change the brain enzyme structure and activity, leading to changes in brain function (Segarra et al., 2011). Future studies need to investigate to what extent diets enriched in fatty acids can induce similar biochemical and functional changes in human brains.

The strengths of this study include: (1) the RCT study design; (2) community-driven, relatively large sample of healthy older adults from five Mediterranean and non-Mediterranean European countries; (3) use of food diary records, which minimize the reliance on memory to assess the usual food intake (Berendsen et al., 2014); (4) extensive baseline and post-intervention cognitive assessments including validated tests; (5) good compliance with the intervention and low overall dropout rates; and (6) the adjustment for multiple potential confounders, including possible confounding related to enrollment in different centers. Additionally, NU-AGE's multicenter recruitment not only allowed for a larger sample size, but also overcame the issue related to the cultural-dependence of Mediterranean-like dietary habits. On the other hand, some limitations must be acknowledged. First, the 1-year duration of the intervention might have been insufficient to detect significant cognitive improvements in the intervention group compared to controls. Indeed, at the follow-up examination, participants in the intervention group showed a tendency toward improved cognitive performance compared to controls, but differences were small and not statistically significant. Future dietary trials, in which real changes in food intake are accomplished, require a longer intervention duration to observe significant effects of cognition. Second, as participants in the control group received a leaflet with national dietary guidelines, they may have changed their habitual dietary behavior closer to the healthier NU-AGE diet. However, this is unlikely as in the control group the adherence to the national dietary guidelines was similar at baseline and follow-up (51.5 and 52.7%, respectively). Third, enrollment of selected volunteers, based on eligibility criteria in NU-AGE trial, might have introduced a selection bias, leading to an underestimation in the strength of the associations. Selective loss to follow-up (dropouts) might have also introduced a second source of bias. Compared to participants, dropouts at follow-up were less healthy and had lower cognitive functioning at baseline, meaning that the observed effect magnitudes could have been underestimated. However, in the mITT analyses (excluding dropouts) the magnitudes of the effects of the NU-AGE diet on cognitive functioning were more similar to the ITT analyses (including dropouts). Fourth, because the NU-AGE intervention included both daily vitamin D supplements and dietary advice, it is difficult to disentangle how much each part contributed to the neurocognitive effects. Finally, potential residual confounding—that is a common concern in all epidemiological (both observational and interventional) studies—cannot be ruled out. Taking into account the above strengths and limitations, we believe that findings from the NU-AGE's individually tailored intervention can be generalized to healthy European older individuals with features similar to NU-AGE.

In summary, an individually tailored NU-AGE dietary intervention that highly adheres to the nutritional principles of the Mediterranean diet could be an effective preventive strategy for cognitive impairment and dementia in countries with different nutritional habits. However, long-duration interventions are needed to confirm the neurocognitive benefits possibly related to the NU-AGE diet. Future research is needed to better understand how long the cognitive benefits can be maintained after the trial is completed, and if it exists, whether there is a ceiling threshold after which no intervention-related cognitive benefit can be observed. Finally, to improve prevention of multi-etiological disorders such as dementia, future trials need to understand whether changes in nutritional behaviors correspond to modification in other lifestyle behaviors (e.g., physical activity and social life) and how these lifestyle multi-components interact with each other in diminishing dementia risk.

## Author contributions

AM: contributed to the conception and design of the current work, data analyses, data interpretation, and drafted the manuscript; ClF: conceived, designed, initiated, and directed NU-AGE; AS: coordinated NU-AGE data collection across centers; LD: initiated; AB: designed and directed the dietary intervention; SF-T: designed, initiated and directed data collection and analyses of biological samples; WX and LF: coordinated the data collection of the cognitive data in NU-AGE; CrF, AB-D, AJ, RG, NM, EC, BP: substantially contributed to the data collection by acquiring or processing data. All authors contributed to interpretation of data, critically revised and approved the final version of this manuscript.

### Conflict of interest statement

The authors declare that the research was conducted in the absence of any commercial or financial relationships that could be construed as a potential conflict of interest.
