# Supplementary material for: Effect of the NU-AGE Diet on Cognitive Functioning in Older Adults: A Randomized Controlled Trial
Source: Front Physiol. 2018 Apr 4;9:349. doi: 10.3389/fphys.2018.00349 (PMC5893841; doi:10.3389/fphys.2018.00349)
Supplement: Supplementary file 1 [file Table1.docx]

**Supplementary Table 1 |** Baseline and follow-up characteristics (means ± SDs) in global and domain-specific cognitive functioning.

|  |  | **Baseline** | | | |  | **Follow-up** | | | |
| --- | --- | --- | --- | --- | --- | --- | --- | --- | --- | --- |
| **Cognitive functioning** | **No. participants with available information** | **Control**  **n=641** |  | **Intervention**  **n=638** | ***p*-value ^a^** |  | **Control**  **n=571** |  | **Intervention**  **n=573** | ***p*-value ^a^** |
| CTS ^b^ | 1241 | 0.03 ± 0.98 |  | -0.05 ± 1.03 | 0.176 |  | 0.30 ± 0.96 |  | 0.24 ± 0.98 | 0.276 |
| Perceptual speed | 1138 | -0.03 ± 0.83 |  | 0.04 ± 0.78 | 0.141 |  | 0.07 ± 0.84 |  | 0.09 ± 0.82 | 0.729 |
| Executive functions | 1243 | 0.03 ± 0.76 |  | -0.06 ± 0.85 | 0.056 |  | 0.17 ± 0.74 |  | 0.09 ± 0.77 | 0.084 |
| Episodic memory | 1243 | 0.02 ± 0.78 |  | -0.03 ± 0.83 | 0.316 |  | 0.19 ± 0.78 |  | 0.15 ± 0.84 | 0.362 |
| Immediate | 1243 | 0.03 ± 0.83 |  | -0.03 ± 0.85 | 0.260 |  | 0.20 ± 0.85 |  | 0.16 ± 0.88 | 0.458 |
| Delay | 1243 | 0.01 ± 0.83 |  | -0.03 ± 0.89 | 0.441 |  | 0.18 ± 0.81 |  | 0.13 ± 0.89 | 0.323 |
| Verbal abilities | 1242 | -0.02 ± 1.02 |  | 0.02 ± 0.98 | 0.473 |  | 0.17 ± 0.91 |  | 0.13 ± 0.95 | 0.520 |
| Constructional praxis | 1242 | -0.03 ± 1.04 |  | 0.03 ± 0.93 | 0.318 |  | -0.11 ± 1.02 |  | -0.13 ± 1.01 | 0.682 |

Abbreviations: CTS, CERAD total score; SDs, standard deviations.

^a^ Two-sample t-test (*p*-value <0.05).

^b^ Higher scores indicate better performance.

**Supplementary Table 2** | Comparisons of demographics, health-related, and cognitive characteristics between the five centers at baseline.

|  | **France** | | **The Netherlands** | | |  | **Italy** | |  | **Poland** | |  | **UK** | | *p* ^c^ |
| --- | --- | --- | --- | --- | --- | --- | --- | --- | --- | --- | --- | --- | --- | --- | --- |
| Baseline  characteristics | **Control** | **Intervention** |  | **Control** | **Intervention** |  | **Control** | **Intervention** |  | **Control** | **Intervention** |  | **Control** | **Intervention** |  |
|  | n = 105 | n = 105 |  | n = 129 | n = 123 |  | n = 152 | n = 146 |  | n = 124 | n = 128 |  | n = 131 | n = 136 |  |
| Age, years | 70.1 ± 3.8 | 70.2 ± 3.8 |  | 71.0 ± 3.9 | 70.9 ± 4.2 |  | 71.9 ± 3.9 | 71.4 ± 3.9 |  | 71.6 ± 3.7 | 71.3 ± 4.0 |  | 71.4 ± 3.9 | 71.4 ± 3.9 | <0.000 |
| Female sex | 47 (44.8) | 59 (56.2) |  | 69 (53.5) | 71 (57.7) |  | 82 (54.0) | 77 (52.7) |  | 71 (57.3) | 74 (57.8) |  | 84 (64.1) | 86 (63.2) | 0.040 |
| Education |  |  |  |  |  |  |  |  |  |  |  |  |  |  |  |
| Elementary | 3 (3.0) | 2 (2.0) |  | 4 (3.1) | 1 (0.8) |  | 16 (11.9) | 12 (8.8) |  | 1 (0.8) | 1 (0.8) |  | 1 (0.8) | 0 (0.0) | <0.000 |
| Secondary | 60 (59.4) | 52 (52.0) |  | 89 (69.0) | 80 (65.6) |  | 84 (62.2) | 92 (67.2) |  | 28 (22.8) | 30 (23.4) |  | 42 (32.1) | 47 (34.6) |  |
| College/university | 38 (37.6) | 46 (46.0) |  | 36 (27.9) | 41 (33.6) |  | 35 (25.9) | 33 (24.1) |  | 94 (76.4) | 97 (75.8) |  | 88 (67.2) | 89 (65.4) |  |
| Frailty status |  |  |  |  |  |  |  |  |  |  |  |  |  |  |  |
| No | 91 (91.9) | 16 (18.4) |  | 97 (76.6) | 94 (79.0) |  | 94 (81.7) | 93 (73.8) |  | 77 (71.3) | 71 (62.3) |  | 107 (87.0) | 105 (83.3) | <0.000 |
| Pre-frail | 8 (8.1) | 138 (24.1)^a^ |  | 28 (22.4) | 25 (21.0) |  | 21 (18.3) | 33 (26.2) |  | 31 (28.7) | 43 (37.7) |  | 16 (13.0) | 21 (16.7) |  |
| MMSE | 28.9 ± 1.24 | 28.9 ± 1.21 |  | 28.1 ± 1.51 | 28.1 ± 1.67 |  | 28.7 ± 1.37 | 27.9 ± 2.69 ^a^ |  | 27.8 ± 1.59 | 27.7 ± 1.80 |  | 28.2 ± 1.87 | 28.7 ± 1.34 ^a^ | <0.000 |
| HbA1c, % | 5.8 ± 0.4 | 5.8 ± 0.4 |  | 6.0 ± 0.4 | 5.9 ± 0.3 |  | 5.7 ± 0.5 | 5.7 ± 0.4 |  | 5.9 ± 0.5 | 5.8 ± 0.4 |  | 5.9 ± 0.5 | 5.8 ± 0.4 | <0.000 |
| BMI, kg/m^2^ | 25.4 ± 3.5 | 25.3 ± 3.4 |  | 26.5 ± 3.4 | 25.5 ± 3.7 ^a^ |  | 27.1 ± 3.9 | 27.5 ± 4.0 |  | 27.5 ± 3.9 | 28.5 ± 4.4 ^a^ |  | 27.5 ± 3.9 | 28.5 ± 4.4 ^a^ |  |
| Underweight [<20] | 5 (4.8) | 2 (1.9) |  | 5 (3.9) | 1 (0.8) |  | 3 (2.0) | 1 (0.7) |  | 0 (0.0) | 1 (0.8) |  | 0 (0.0) | 4 (2.9) | <0.000 |
| Normal [20-25] | 45 (43.3) | 55 (52.4) |  | 34 (26.4) | 62 (50.4) |  | 41 (27.2) | 40 (27.4) |  | 36 (29.0) | 24 (18.8) |  | 45 (34.4) | 44 (32.4) | <0.000 |
| Overweight [25-30] | 41 (39.4) | 38 (36.2) |  | 69 (53.5) | 45 (36.6) ^a^ |  | 76 (50.3) | 74 (50.7) |  | 54 (43.6) | 62 (48.4) |  | 64 (48.9) | 61 (44.9) |  |
| Obese [≥30] | 13 (12.5) | 10 (9.5) |  | 21 (16.3) | 15 (12.2) |  | 31 (20.5) | 31 (21.2) |  | 34 (27.4) | 41 (32.0) |  | 22 (16.8) | 27 (20.0) |  |
|  |  |  |  |  |  |  |  |  |  |  |  |  |  |  |  |
| **Medical conditions** |  |  |  |  |  |  |  |  |  |  |  |  |  |  |  |
| Sensorial | 81 (80.2) | 76 (76.8) |  | 52 (40.3) | 55 (44.7) |  | 49 (36.3) | 40 (29.0) |  | 42 (33.9) | 79 (61.7) |  | 93 (71.0) | 93 (68.4) | <0.000 |
| Hypertension | 33 (32.7) | 33 (33.0) |  | 44 (34.1) | 39 (31.7) |  | 62 (45.9) | 74 (53.6) |  | 70 (56.5) | 82 (64.1) |  | 40 (30.5) | 37 (27.2) | <0.000 |
| Hypercholesterolemia | 34 (34.7) | 28 (28.3) |  | 35 (27.1) | 29 (23.6) |  | 62 (45.9) | 68 (49.3) |  | 50 (40.3) | 46 (35.9) |  | 25 (19.1) | 34 (25.0) | <0.000 |
| Cardiovascular | 32 (31.7) | 39 (39.0) |  | 20 (15.5) | 26 (21.1) |  | 51 (37.8) | 56 (40.6) |  | 61 (49.2) | 49 (38.3) |  | 16 (12.2) | 20 (14.7) | <0.000 |
| Stroke/TIA | 1 (1.0) | 0 (0.0) |  | 2 (1.6) | 3 (2.4) |  | 3 (2.2) | 3 (2.2) |  | 3 (2.4) | 4 (3.1) |  | 3 (2.3) | 3 (2.2) | 0.522 |
| Diabetes | 5 (5.0) | 4 (4.0) |  | 6 (4.7) | 3 (2.4) |  | 8 (5.9) | 9 (6.5) |  | 10 (8.1) | 11 (8.6) |  | 4 (3.1) | 3 (2.2) | 0.027 |
| Neurological | 1 (1.0) | 2 (2.0) |  | 0 (0.0) | 3 (2.4) |  | 9 (6.7) | 9 (6.5) |  | 2 (1.6) | 4 (3.1) |  | 2 (1.5) | 1 (0.7) | <0.000 |
| Mental health | 5 (5.1) | 14 (14.1) ^a^ |  | 2 (1.6) | 3 (2.4) |  | 13 (9.6) | 16 (11.6) |  | 3 (2.4) | 6 (4.7) |  | 3 (2.3) | 5 (3.7) | <0.000 |
| Hyperthyroid | 1 (1.0) | 1 (1.0) |  | 1 (0.8) | 1 (0.8) |  | 0 (0.0) | 1 (0.7) |  | 2 (1.6) | 5 (3.9) |  | 0 (0.0) | 1 (0.7) | 0.043 |
| Hypothyroid | 16 (16.0) | 18 (18.0) |  | 5 (3.9) | 8 (6.5) |  | 15 (11.1) | 12 (8.7) |  | 9 (7.3) | 16 (12.5) |  | 13 (9.9) | 9 (6.6) | 0.001 |
| Osteoporosis | 13 (13.4) | 13 (13.3) |  | 7 (5.4) | 19 (15.5)^a^ |  | 20 (14.8) | 13 (9.4) |  | 26 (21.0) | 26 (20.3) |  | 2 (1.5) | 8 (5.9) | <0.000 |
| Arthritis | 23 (24.2) | 28 (28.3) |  | 20 (15.5) | 24 (19.5) |  | 58 (43.0) | 62 (44.9) |  | 41 (33.1) | 40 (31.3) |  | 51 (38.9) | 48 (35.3) | <0.000 |
|  |  |  |  |  |  |  |  |  |  |  |  |  |  |  |  |
| **Cognitive functioning ^b^** |  |  |  |  |  |  |  |  |  |  |  |  |  |  |  |
| CTS | 0.22 ± 0.93 | 0.18 ± 0.90 |  | 0.40 ± 0.86 | 0.30 ± 0.97 |  | 0.06 ± 1.05 | -0.13 ± 1.02 |  | 0.50 ± 0.92 | 0.35 ± 1.02 |  | 0.31 ± 1.00 | 0.49 ± 0.85 | <0.000 |
| Perceptual speed | 0.67 ± 0.77 | 0.56 ± 0.79 |  | 0.26 ± 0.71 | 0.21 ± 0.67 |  | 0.14 ± 0.82 | 0.35 ± 0.87^a^ |  | -0.10 ± 0.83 | -0.05 ± 0.73 |  | -0.50 ± 0.63 | -0.49 ± 0.65 | <0.000 |
| Executive function | -0.10 ± 0.82 | -0.07 ± 0.77 |  | 0.44 ± 0.57 | 0.36 ± 0.71 |  | 0.28 ± 0.70 | 0.16 ± 0.68 |  | 0.05 ± 0.71 | -0.21 ± 0.86 ^a^ |  | 0.12 ± 0.79 | 0.15 ± 0.71 | <0.000 |
| Episodic memory | 0.08 ± 0.70 | 0.03 ± 0.72 |  | -0.22 ± 0.66 | -0.19 ± 0.74 |  | 0.19 ± 0.84 | -0.03 ± 0.86 ^a^ |  | 0.45 ± 0.77 | 0.28 ± 0.88 |  | 0.48 ± 0.72 | 0.61 ± 0.73 | <0.000 |
| Immediate | 0.12 ± 0.74 | 0.04 ± 0.75 |  | -0.22 ± 0.73 | -0.18 ± 0.76 |  | 0.14 ± 0.92 | -0.01 ± 0.91 |  | 0.50 ± 0.85 | 0.33 ± 0.91 |  | 0.50 ± 0.79 | 0.60 ± 0.80 | <0.000 |
| Delay | 0.05 ± 0.75 | 0.03 ± 0.79 |  | -0.21 ± 0.70 | -0.20 ± 0.79 |  | 0.24 ± 0.86 | -0.05 ± 0.94 ^a^ |  | 0.39 ± 0.78 | 0.23 ± 0.92 |  | 0.46 ± 0.77 | 0.63 ± 0.74 | <0.000 |
| Verbal abilities | 0.13 ± 0.97 | 0.02 ± 0.93 |  | -0.03 ± 1.03 | -0.01 ± 0.95 |  | 0.33 ± 0.75 | 0.27 ± 0.73 |  | 0.25 ± 0.85 | -0.03 ± 1.37 |  | 0.20 ± 0.90 | 0.36 ± 0.59 | <0.000 |
| Constructional praxis | -0.13 ± 0.91 | -0.22 ± 1.1 |  | -0.59 ± 1.22 | -0.67 ± 1.11 |  | -0.39 ± 1.11 | -0.49 ± 1.01 |  | 0.47 ± 0.51 | 0.44 ± 0.53 |  | 0.15 ± 0.80 | 0.28 ± 0.62 | <0.000 |

Abbreviations: CTS, CERAD total score; MMSE, Mini Mental State Examination;

Data are presented as proportion [n (%)] or mean ± standard deviations (SDs).

^a^ p value <0.05 for between–groups (control vs. intervention) comparisons within each country. Chi-square test was used to compare proportions and two–sample t–test to compare means ± SDs

^b^ Higher scores indicate better performance

^c^ *p*-value <0.05 for overall comparisons between enrollment countries.
